# Safety and clinical activity of JNJ-78306358, a human leukocyte antigen-G (HLA-G) x CD3 bispecific antibody, for the treatment of advanced stage solid tumors

**DOI:** 10.1007/s00262-024-03790-7

**Published:** 2024-08-06

**Authors:** Ravit Geva, Maria Vieito, Jorge Ramon, Ruth Perets, Manuel Pedregal, Elena Corral, Bernard Doger, Emiliano Calvo, Jorge Bardina, Elena Garralda, Regina J. Brown, James G. Greger, Shujian Wu, Douglas Steinbach, Tsun-Wen Sheena Yao, Yu Cao, Josh Lauring, Ruchi Chaudhary, Jaymala Patel, Bharvin Patel, Victor Moreno

**Affiliations:** 1 Sourasky Medical Center, Tel-Aviv university, Tel-Aviv, Israel; 2 Vall d’Hebron Institute of Oncology (VHIO), Barcelona, Spain; 3 START Madrid-CIOCC, Centro Integral Oncológico Clara Campal, Madrid, Spain; 4 Rambam Medical Center, and Technion–Israel Institute of Technology, Haifa, Israel; 5 START Madrid-FJD, Hospital Fundacion Jimenez Diaz, Madrid, Spain; 6 Janssen Research and Development, Spring House, PA USA; 7 Janssen Research and Development, Horsham, PA USA; 8 Janssen Research and Development, Raritan, NJ USA

**Keywords:** Cytokine release syndrome, Dose escalation study, Human leukocyte antigen-G, JNJ-78306358, Phase 1, Solid tumors

## Abstract

**Background:**

JNJ-78306358 is a bispecific antibody that redirects T cells to kill human leukocyte antigen-G (HLA-G)-expressing tumor cells. This dose escalation study evaluated the safety, pharmacokinetics, pharmacodynamics, and preliminary antitumor activity of JNJ-78306358 in patients with advanced solid tumors.

**Methods:**

Adult patients with metastatic/unresectable solid tumors with high prevalence of HLA-G expression were enrolled. Dose escalation was initiated with once-weekly subcutaneous administration with step-up dosing to mitigate cytokine release syndrome (CRS).

**Results:**

Overall, 39 heavily pretreated patients (colorectal cancer: n = 23, ovarian cancer: n = 10, and renal cell carcinoma: n = 6) were dosed in 7 cohorts. Most patients (94.9%) experienced ≥ 1 treatment-emergent adverse events (TEAEs); 87.2% had ≥ 1 related TEAEs. About half of the patients (48.7%) experienced CRS, which were grade 1/2. Nine patients (23.1%) received tocilizumab for CRS. No grade 3 CRS was observed. Dose-limiting toxicities (DLTs) of increased transaminases, pneumonitis and recurrent CRS requiring a dose reduction were reported in 4 patients, coinciding with CRS. No treatment-related deaths reported. No objective responses were noted, but 2 patients had stable disease > 40 weeks. JNJ-78306358 stimulated peripheral T cell activation and cytokine release. Anti-drug antibodies were observed in 45% of evaluable patients with impact on exposure. Approximately half of archival tumor samples (48%) had expression of HLA-G by immunohistochemistry.

**Conclusion:**

JNJ-78306358 showed pharmacodynamic effects with induction of cytokines and T cell activation. JNJ-78306358 was associated with CRS-related toxicities including increased transaminases and pneumonitis which limited its dose escalation to potentially efficacious levels.

*Trial registration number* ClinicalTrials.gov (No. NCT04991740).

**Supplementary Information:**

The online version contains supplementary material available at 10.1007/s00262-024-03790-7.

## Introduction

Tumor-associated antigens (TAA) are neo-expressed or upregulated on tumor cells, while their expression on normal cells is restricted. This differential expression pattern makes TAAs attractive targets for immune-targeting modalities such as CD3-redirecting bispecific antibodies [1]. The bispecific antibodies are engineered to simultaneously bind to a TAA on the tumor cell and to T cell markers such as CD3. This dual binding mechanism bridges the gap between the immune system and the tumor, facilitating the engagement of cytotoxic T cells (CTLs) to kill the tumor cells [2, 3]. Bispecific-antibody-based strategies for targeting T cells have gained relevance with the regulatory approval of several bispecific molecules for diverse tumors [3–5].

Human leukocyte antigen-G (HLA-G) is a non-classical major histocompatibility complex class 1 molecule with a crucial role in maintaining fetal-maternal immune tolerance [6]. HLA-G exhibits limited expression in normal tissues but is expressed in various types of human cancers, where it may play a role in immune system evasion. HLA-G expression has been associated with several malignancies, including breast cancer, colorectal cancer, ovarian cancer, and lung cancer, among others [7, 8]. Particularly, HLA-G expression has been associated with advanced disease stages, increased tumor metastasis, worse prognosis and reduced disease-free survival [9–11]. The immune-suppressive function of HLA-G supports its role in tumor development and progression. HLA-G functions as an immune checkpoint ligand that interacts with inhibitory receptors, including immunoglobulin-like transcript 2 (ILT2) and immunoglobin-like transcript 4 (ILT4) expressed on monocytes, neutrophils, B and T lymphocytes, natural killer cells, dendritic cells and myeloid-derived suppressive cells [12].

JNJ-78306358 is a first-in-class immunoglobulin (Ig)G1 bispecific antibody developed for the treatment of advanced solid tumors that express HLA-G. JNJ-78306358 simultaneously binds to the α3 domain of HLA-G isoforms on tumor cells and to the CD3 receptor complex on T cells [12], mediating immune synapse formation and tumor cell killing by cytotoxic T cells, while mitigating the immunosuppressive tumor microenvironment [13].

In vitro studies demonstrated that JNJ-78306358 exhibits peripheral blood mononuclear cell- and T cell- based cytotoxicity against endogenous membrane HLA-G-expressing tumor cell lines with increased potency with increasing HLA-G expression. No activity was observed against cancer cells lacking HLA-G membrane expression, underscoring its specificity for antigen-expressing tumor cells. JNJ-78306358 also demonstrated T cell engagement in vitro, including T cell proliferation and cytokine release [13]. In murine patient-derived and human cell line-derived xenograft models, JNJ-78306358 exhibited HLA-G-expression-dependent antitumor activity. A dose dependent increase in CD4 + and CD8 + T cell infiltration into implanted tumors was observed, leading to complete tumor regression [13].

Despite recent advances in T cell-based immunotherapies [14–16], alternative therapies for the treatment of metastatic disease after disease progression in tumor types such as renal cell carcinoma (RCC), ovarian cancer (OC), and colorectal cancer (CRC) are limited. The distinctive mode of action of JNJ-78306358 gives it the potential to be effective against tumor types often characterized as “immunologically cold”, such as OC and CRC, as well as those that exhibit recurrence despite immune checkpoint inhibitor therapies, like RCC. Based on internal immunohistochemistry (IHC) data, RCC, OC and CRC emerged as tumor types with the highest prevalence of HLA-G expression.

The current report presents the findings from an open label, phase 1, dose escalation study designed to determine the recommended phase 2 dose (RP2D) based on safety, and evaluate pharmacokinetics (PK), pharmacodynamics (PD), immunogenicity, and preliminary antitumor activity of JNJ-78306358 in patients with advanced stage solid tumors having high prevalence of HLA-G protein expression.

## Methods

### Study design and patients

This was an open label, phase 1 dose finding study conducted between October 2021 and February 2023 at 5 sites in Spain and Israel that evaluated JNJ-78306358 in participants with metastatic or unresectable solid tumor types with a high prevalence of HLA-G expression. The study was to be conducted in 2 parts: dose escalation (Part 1) and dose expansion (Part 2). The primary objectives of this study were to determine the recommended phase 2 dose(s) (RP2Ds) of JNJ 78306358 and to determine the safety of JNJ-78306358 at the RP2D(s) by evaluating the incidence and severity of adverse events (AEs), including dose-limiting toxicity (DLT). Secondary objectives were to assess the pharmacokinetics (PK), immunogenicity, and preliminary antitumor activity of JNJ-78306358.

**Fig. 1:**
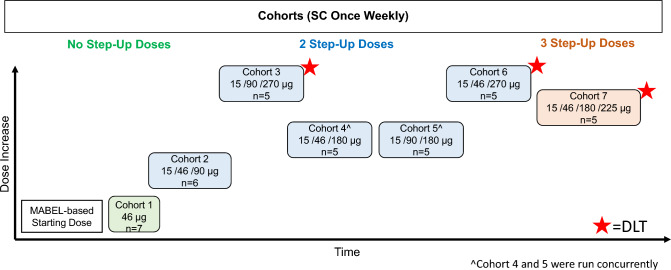
Dose escalation. Step-up doses and the treatment dose are shown for each cohort. *DLT* dose-limiting toxicities, *MABEL* minimum anticipated biological effect level, *SC* subcutaneous

Dose escalation was initiated at 46 µg administered subcutaneously (SC) once a week and subsequent dose levels were determined using a continuous reassessment method based on a Bayesian regression model (Fig. 1). To mitigate cytokine release syndrome (CRS) potential, all patients received their first dose of study drug premedicated with a corticosteroid, antipyretic and antihistamine. The corticosteroid could be weaned for subsequent doses. Step-up doses were implemented to mitigate CRS at the treatment dose for the cohort. Treatment continued until unequivocal radiographic or clinical disease progression, unacceptable toxicity or withdrawal of consent occurred.

Key inclusion criteria were the following: adults (≥ 18 years) with histologically or cytologically confirmed metastatic unresectable solid tumor types with high prevalence of HLA-G expression, including RCC, OC, and CRC. RCC patients were required to have progression after treatment with an antiangiogenic agent and an immune checkpoint inhibitor. CRC patients were required to have progression after treatment with at least 2 lines of therapy including fluoropyrimidine, oxaliplatin, and irinotecan. In addition, prior treatment with anti-PD1 antibody was required for microsatellite instability high (MSI-H) CRC. OC patients were required to have progression after treatment with at least 2 lines of therapy, including at least 1 line with platinum. Patients were to have measurable or evaluable disease, an Eastern Cooperative Oncology Group (ECOG) performance status of grade 0 or 1 and adequate organ function.

Key exclusion criteria were the following: active central nervous system involvement, prior treatment with HLA-G-targeted therapy, prior anti-cytotoxic T-lymphocyte antigen-4 or anti-programmed cell death receptor-1 therapy within 6 weeks or other anticancer therapy within 14 days before the first dose of study drug, clinically significant pulmonary compromise, autoimmune or inflammatory disease requiring systemic steroids or other immunosuppressive agents within 1 year of study start.

Safety was assessed by physical examinations, neurologic evaluations, ECOG performance status, vital signs, electrocardiograms, clinical safety laboratory tests, pregnancy testing, and monitoring for AEs, including DLTs. Treatment-emergent adverse events (TEAEs) were graded per National Cancer Institute-Common Terminology Criteria for Adverse Events [NCI-CTCAE] version 5.0, except for CRS and immune effector cell-associated neurotoxicity syndrome, which was graded according to the American Society for Transplantation and Cellular Therapy (ASTCT) guidelines [17]. DLTs were monitored during the first 21 days after the first full treatment dose and were defined as certain grade ≥ 3 hematologic toxicities and any non-hematological toxicity of grade ≥ 3 or that resulted in treatment discontinuation with certain exceptions (Table S2).

### Efficacy

Computed tomography (CT) or magnetic resonance imaging (MRI) of the chest, abdomen and pelvis was performed every 8 weeks for the first 24 weeks, and then every 12 weeks while on treatment. CA-125 was obtained in participants with OC every 4 weeks. Objective response rate (ORR) was evaluated by the investigator using the Response Evaluation Criteria in Solid Tumors (RECIST) version 1.1 and Gynecological Cancer InterGroup (GCIG) CA 125 response criteria (OC only).

### PK, PD and immunogenicity

Blood samples were collected for measurement of PK, PD (serum cytokine and peripheral blood immunophenotyping), and anti-drug antibodies (ADA). JNJ-78306358 serum concentrations were determined using an electrochemiluminescence-based immunoassay (ECLIA) on the Meso Scale Discovery platform by Janssen R&D. Serum cytokines collected pre- and post-JNJ-78306358 treatment as well as during grade ≥ 2 CRS were measured by a Meso Scale Discovery (MSD) assay using the MSD-V-PLEX Plus Pro-inflammatory Panel 1 Human Kit (Cat# K15049G) at CellCarta using an MSD Sector ® S600 analyzer. Fold change was normalized to baseline (pre-dose) values. Immunophenotyping was performed on whole blood using the TBNK Panel (CD3, CD4, CD8, CD16, CD19, CD45, CD56) and the T memory and Activation Panel (CD3, CD4, CD8, CD25, CD45, CD45RO, CD127, CCR7, FoxP3, Ki67, HLA-DR, CD278, CD38, CD279) at CellCarta and analyzed by the BD LSR Fortessa X-20 analyzer (Table S1). Fold change of median fluorescent intensity (MdFI) and % positivity were normalized to baseline (pre-dose) values. IHC was performed at CellCarta using the 4H84 monoclonal antibody (Abcam, Cat# ab52455) on Ventana Benchmark Ultra platform. Global H-score in the tumors was reported.

Levels of soluble HLA-G (sHLA-G) were assessed from baseline (pre-dose) serum samples to understand the risk of target-mediated drug disposition effect. Soluble HLA-G levels were measured at Janssen Research & Development using an MSD assay with JNJ-78306358 and 4H84 mAb as capturing and detecting antibodies respectively.

### Statistical analysis

No formal statistical hypothesis was tested in this study. Dose escalation was guided by a modified continual reassessment method (mCRM) based on a Bayesian logistic regression model with overdose control such that the posterior probability of the DLT rate was within the target toxicity interval of 33%.

Descriptive statistics were used for demographic, safety, efficacy, PK, and biomarker data by dose level and time, and were summarized as number of observations, standard deviation, coefficient of variation, median, and range. Categorical data were summarized using frequency counts and percentages. ORR was defined as the proportion of patients who achieved a partial response or complete response according to RECIST v1.1. All assessments in this study were conducted on ‘all treated analysis set’ which included patients who received at least 1 dose of JNJ-78306358.

## Results

Overall, 39 patients (23 with CRC, 10 with OC, and 6 with RCC) were enrolled and treated once-weekly in 7 cohorts in dose escalation (Part 1). The study did not proceed to dose expansion (Part 2). All 39 patients (100.0%) discontinued the study treatment. The most common reasons were progressive disease (82.1%) and death (5.1%), none considered related to JNJ-78306358. One (2.6%) patient discontinued study treatment due to TEAEs.

Median age of patients was 62 years (range: 39–80 years); the majority were women (61.5%). Patients were heavily pretreated: most patients (53.8%) received ≥ 4 prior lines of systemic therapy for metastatic disease (Table 1). The median duration of JNJ-78306358 exposure including step-up dosing was 62 days (range: 1–345 days).

**Table 1:** Baseline demographics and disease characteristics

	JNJ-78306358 Q1W SC dosing							Total (N = 39)
	46 µg (n = 7)	15–46–90 µg (n = 6)	15–90–270 µg (n = 5)	15–46–180 µg (n = 5)	15–90–180 µg (n = 5)	15–46–270 µg (n = 5)	15–46–180–225 µg (n = 6)	
Age, mean (SD), y	60.6 (9.98)	64.3 (7.12)	62.8 (15.99)	64.4 (11.44)	59.0 (10.84)	59.2 (9.78)	58.8 (2.86)	61.3 (9.59)
Sex, n (%)								
Women,	5 (71.4)	5 (83.3)	4 (80.0)	2 (40.0)	1 (20.0)	5 (100.0)	2 (33.3)	24 (61.5)
Race, n (%)								
White	6 (85.7)	6 (100.0)	4 (80.0)	5 (100.0)	5 (100.0)	5 (100.0)	6 (100.0)	37 (94.9)
Unknown	1 (14.3)	0	1 (20.0)	0	0	0	0	2 (5.1)
ECOG status, n (%)								
0	7 (100.0)	4 (66.7)	4 (80.0)	3 (60.0)	3 (60.0)	1 (20.0)	5 (83.3)	27 (69.2)
1	0	2 (33.3)	1 (20.0)	2 (40.0)	2 (40.0)	4 (80.0)	1 (16.7)	12 (30.8)
Tumor type, n (%)								
RCC	1 (14.3)	1 (16.7)	1 (20.0)	1 (20.0)	0	1 (20.0)	1 (16.7)	6 (15.4)
CRC	3 (42.9)	2 (33.3)	4 (80.0)	3 (60.0)	5 (100.0)	3 (60.0)	3 (50.0)	23 (59.0)
OC	3 (42.9)	3 (50.0)	0	1 (20.0)	0	1 (20.0)	2 (33.3)	10 (25.6)
Prior systemic therapies, n (%)								
1	0	0	1 (20.0)	0	1 (20.0)	0	0	2 (5.1)
2	2 (28.6)	0	1 (20.0)	0	2 (40.0)	2 (40.0)	0	7 (17.9)
3	0	3 (50.0)	1 (20.0)	1 (20.0)	2 (40.0)	1 (20.0)	1 (16.7)	9 (23.1)
≥ 4	5 (71.4)	3 (50.0)	2 (40.0)	4 (80.0)	0	2 (40.0)	5 (83.3)	21 (53.8)

*CRC* colorectal cancer; *ECOG* Eastern Cooperative Oncology Group; *OC* ovarian cancer; *Q1W* once every week; *RCC* renal cell carcinoma; *SC* subcutaneous; *SD* standard deviation

### Safety

Overall, 37 (94.9%) patients experienced ≥ 1 TEAE (Table 2). The most commonly reported TEAEs (> 25%) were CRS (48.7%), injection site erythema, alanine aminotransferase (ALT) increased, aspartate aminotransferase (AST) increased (38.5%, each), fatigue (35.9%), and abdominal pain (25.6%). A majority of the patients (64.1%) had an injection site reaction with injection site erythema as the most common reaction.

**Table 2:** Summary of treatment-emergent adverse events

n (%)	JNJ-78306358 Q1W SC dosing							Total (N = 39)
	46 µg (n = 7)	15–46–90 µg (n = 6)	15–90–270 µg (n = 5)	15–46–180 µg (n = 5)	15–90–180 µg (n = 5)	15–46–270 µg (n = 5)	15–46–180–225 µg (n = 6)	
**≥ 1 TEAEs**	7 (100)	6 (100)	5 (100)	5 (100)	4 (80.0)	5 (100)	5 (100)	37 (94.9)
**Most common TEAEs (> 25% in total group)**								
CRS	2 (28.6)	1 (16.7)	5(100)	3 (60.0)	2 (40.0)	3 (60.0)	3 (50.0)	19(48.7)
Injection site erythema	1 (14.3)	3 (50.0)	4 (80.0)	1 (20.0)	2 (40.0)	2 (40.0)	2 (33.3)	15(38.5)
ALT increased	0	1 (16.7)	5(100)	4 (80.0)	2 (40.0)	2 (40.0)	1 (16.7)	15 (38.5)
AST increased	2 (28.6)	1 (16.7)	4 (80.0)	4 (80.0)	2 (40.0)	2 (40.0)	0	15(38.5)
Fatigue	4 (57.1)	2 (33.3)	3 (60.0)	2 (40.0)	0	1 (20.0)	2 (33.3)	14(35.9)
Abdominal pain	2 (28.6)	3 (50.0)	1 (20.0)	1 (20.0)	0	3 (60.0)	0	10(25.6)
**Serious TEAEs**	3 (42.9)	4 (66.7)	2 (40.0)	2 (40.0)	1 (20.0)	5 (100.0)	3 (50.0)	20 (51.3)
**Most common serious TEAEs (> 5% in total group)**								
CRS	1 (14.3)	0	1 (20.0)	0	0	1 (20.0)	3 (50.0)	6 (15.4)
Hyperbilirubinemia	1 (14.3)	0	0	1 (20.0)	0	0	0	2 (5.1)
Back pain	0	1 (16.7)	0	0	0	1 (20.0)	0	2 (5.1)
**Grade ≥ 3 TEAEs**	4 (57.1)	5 (83.3)	2 (40.0)	4 (80.0)	1 (20.0)	5 (100.)	3 (50.0)	24 (61.5)
**Most common grade ≥ 3 TEAEs (> 5% in total group)**								
Back pain	1 (14.3)	2 (33.3)	0	0	0	1 (20.0)	0	4 (10.3)
Anemia	1 (14.3)	0	0	0	1 (20.0)	0	0	2 (5.1)
Lymphopenia	0	1 (16.7)	1 (20.0)	0	0	0	0	2 (5.1)
Neutropenia	1 (14.3)	0	0	0	0	0	1 (16.7)	2 (5.1)
Pneumonia	0	0	0	1 (20.0)	0	1 (20.0)	0	2 (5.1)
Pulmonary embolism	0	0	0	0	1 (20.0)	1 (20.0)	0	2 (5.1)
ALT increased	0	0	0	1 (20.0)	0	1 (20.0)	0	2 (5.1)
Hypertension	1 (14.3)	1 (16.7)	0	0	0	0	0	2 (5.1)
**TEAEs leading to discontinuation**	0	1 (16.7)	0	0	0	0	2 (33.3)	3 (7.7)
Lymphangiosis carcinomatosa	0	0	0	0	0	0	1 (16.7)	1 (2.6)
Hemiparesis	0	1 (16.7)	0	0	0	0	0	1 (2.6)
Pneumonitis	0	0	0	0	0	0	1 (16.7)	1 (2.6)

*ALT* alanine aminotransferase; *AST* aspartate aminotransferase; *CRS* cytokine release syndrome; *TEAE* treatment-emergent adverse event; *SC* subcutaneous; *Q1W* once every week

Twenty-four (61.5%) patients experienced grade ≥ 3 TEAEs (Table 2). Serious TEAEs occurred in 20 (51.3%) patients, the most common being CRS (15.4%). TEAEs leading to treatment discontinuation occurred in 3 patients: lymphangiosis carcinomatosa, hemiparesis and pneumonitis (2.6%, each), of which pneumonitis was considered related to study treatment (Table 3). Nine (23.1%) patients died during the study. The most common cause of death was progressive disease (n = 5, 12.8%). Three (7.7%) patients died during the study due to TEAEs of cholangitis infective, sepsis, and lymphangiosis carcinomatosa (1 patient, each); none of the deaths were considered treatment-related. One patient died during the follow-up phase due to unknown causes after discontinuing treatment due to progressive disease.

**Table 3:** Related treatment-emergent adverse events

n (%)	JNJ-78306358 Q1W SC dosing							Total (N = 39)
	46 µg (n = 7)	15–46–90 µg (n = 6)	15–90–270 µg (n = 5)	15–46–180 µg (n = 5)	15–90–180 µg (n = 5)	15–46–270 µg (n = 5)	15–46–180–225 µg (n = 6)	
**≥ 1 related TEAEs**	7 (100)	4 (66.7)	5 (100)	5 (100)	4 (80.0)	4 (80.0)	5 (83.3)	34 (87.2)
**Most common related TEAEs (> 20% in total group)**								
CRS	2 (28.6)	1 (16.7)	5 (100)	3 (60.0)	2 (40.0)	3 (60.0)	3 (50.0)	19 (48.7)
Injection site erythema	1 (14.3)	3 (50.0)	4 (80.0)	1 (20.0)	2 (40.0)	2 (40.0)	2 (33.3)	15 (38.5)
ALT increased	0	1 (16.7)	5 (100)	4 (80.0)	1 (20.0)	2 (40.0)	1 (16.7)	14 (35.9)
AST increased	1 (14.3)	1 (16.7)	4 (80.0)	4 (80.0)	1 (20.0)	2 (40.0)	0	13 (33.3)
**Related TEAEs leading to discontinuation**	0	0	0	0	0	0	1 (16.7)	1 (2.6)
Pneumonitis	0	0	0	0	0	0	1 (16.7)	1 (2.6)
**Related serious TEAEs**	1 (14.3)	1 (16.7)	1 (20.0)	1 (20.0)	0	1 (20.0)	3 (50.0)	8 (20.5)
CRS	1 (14.3)	0	1 (20.0)	0	0	1 (20.0)	3 (50.0)	6 (15.4)
Hypertransaminasemia	0	0	0	0	0	0	1 (16.7)	1(2.6)
ALT increased	0	1 (16.7)	0	0	0	0	0	1 (2.6)
Oxygen saturation decreased	0	0	0	1 (20.0)	0	0	0	1 (2.6)
Flank pain	0	0	0	1 (20.0)	0	0	0	1 (2.6)
Pneumonitis	0	0	0	0	0	0	1 (16.7)	1 (2.6)

*ALT* alanine aminotransferase; *AST* aspartate aminotransferase; *CRS* cytokine release syndrome; *TEAE* treatment-emergent adverse event; *SC* subcutaneous; *Q1W* once every week

The main safety concern was CRS. Of the 19 (48.7%) patients who had ≥ 1 CRS event, 9 (23.1%) had concurrent hypotension (grade 2) and 9 (23.1%) were treated with tocilizumab. No grade ≥ 3 CRS was observed. Two grade 2 CRS events (28.6%) occurred at the starting dose of 46 µg. Step-up dosing using a lower dose of 15 ug and 46 ug or 90 ug was implemented for subsequent cohorts. Most CRS events occurred after the first treatment dose and resolved within 24 h. There was one recurrent CRS that required a dose reduction and was considered a DLT. Three patients had more than one CRS event including one patient with grade 1 CRS after the step-up doses and the first treatment dose.

### Dose-limiting toxicities

Four patients (10.3%) experienced one or more DLTs, and all of them experienced a CRS event before the DLTs: (i) Hypertransaminasemia (grade 3; JNJ-78306358 15-90-270 ug SC QW cohort), (ii) Recurrent CRS (grade 2 followed by grade 1, requiring dose reduction; JNJ-78306358 15-90-270 ug SC QW cohort), (iii) Increased ALT (grade 3; JNJ-78306358 15-46-270 ug SC QW cohort), and (iv) Pneumonitis (grade 3; JNJ-78306358 15-46-180 ug SC QW cohort).

### Efficacy

**Fig. 2:**
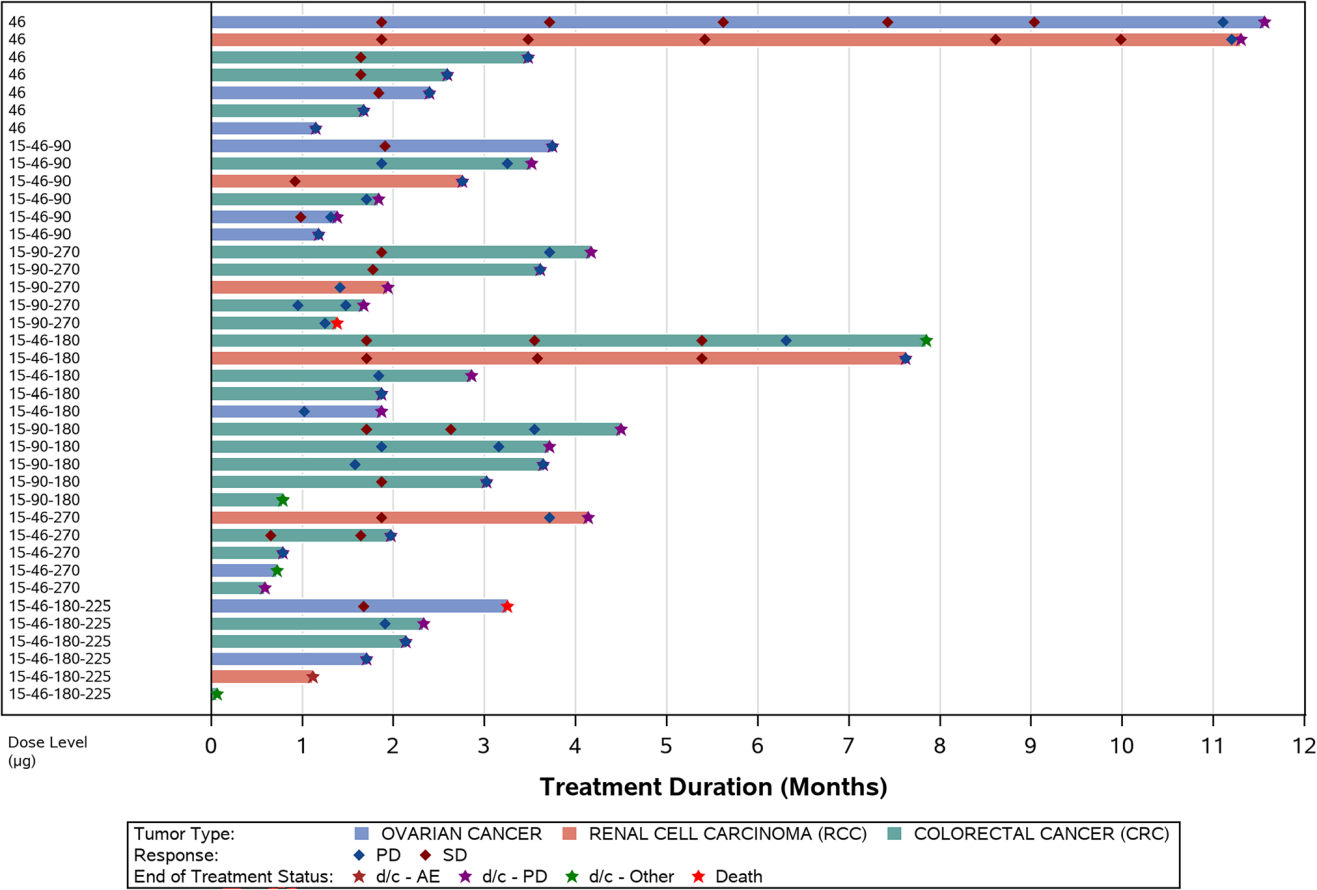
Response and time-on-treatment arranged by cohort; all treated analysis set. *AE* adverse events, *d/c* discontinuation, *PD* progressive disease, *SD* stable disease

There were no objective responses per RECIST Version 1.1 or GCIG CA 125 criteria during the study. Seventeen of the 34 response evaluable patients showed disease stabilization (Figure S1) and 2 patients had stable disease for > 40 weeks (Fig. 2). One participant with OC had a 42% reduction in CA 125.

Of the response evaluable patients, 4 of 5 RCC patients, 5 of 9 OC patients and 8 of 20 CRC patients had SD as their best response.

### Pharmacokinetics

**Fig. 3:**
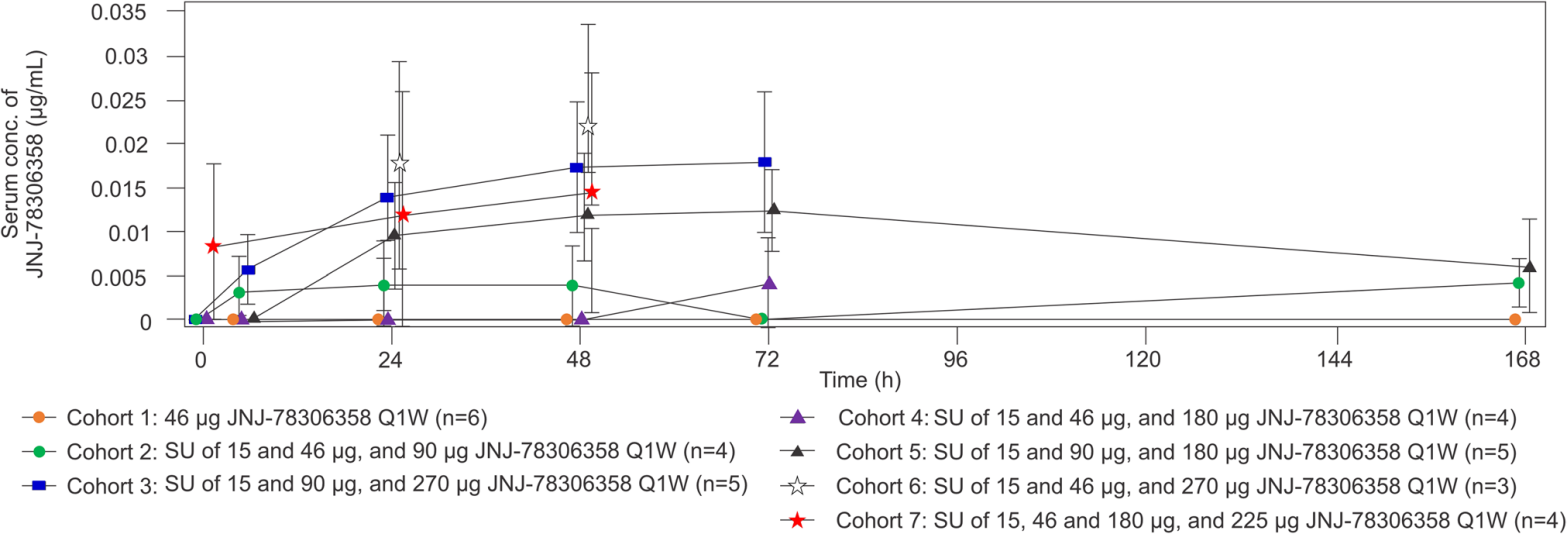
Mean serum concentration–time profiles of JNJ-78306358 after first treatment dose administration. *Conc.* Concentration, *Q1W* once every week, *SC* subcutaneous, *SU* step-up

Dose normalized PK parameters were comparable across all cohorts (Fig. 3). JNJ-78306358 exposure levels (peak concentrations and area under the curve) increased with dose level. Elimination half-life (t_1/2_) was not evaluable. Following weekly SC administration of JNJ-78306358 in different cohorts, Dose 1 mean C_max_, AUC_72h_, and AUC_last_ ranged for cohorts 3–7 between 0.00760–0.0221 μg/mL, 0.661–1.83 μg.h./mL, and 0.722–1.93 μg.h./mL, respectively.

### Immunogenicity

ADA were identified in a number of patients within 7 weeks of treatment initiation. Therefore, patients were considered immunogenicity-evaluable if they tested positive on one sample at any time point (including the end of treatment sample) or if samples tested negative for > 7 total weeks after JNJ-78306358 administration. Of the 27 immunogenicity-evaluable patients, 17 (62.9%) were positive for ADA. ADA positivity and ADA titer did not appear to be dependent on dose. Neutralizing antibodies were detected in 11 of 12 ADA positive samples.

In most cases, ADA was associated with loss of exposure at the same timepoint in which ADA was detected or at a subsequent timepoint. There was no clear association between ADA and infusion-related reactions or CRS. Due to the limited dose escalation and serum drug concentrations falling below the limit of quantification in nearly all ADA positive patients, the impact of antibodies to JNJ-78306358 on PK could not be fully quantified.

### Soluble HLA-G

Baseline sHLA-G levels were detectable in 6/38 patient samples (> LLOQ of 0.015–0.131 ng/mL) with levels ranging from 0.036 to 0.642 ng/mL. Given the low levels of sHLA-G at baseline and the use of JNJ-78306358 as the capture antibody, no evaluation of sHLA-G post-dosing was conducted. No association between sHLA-G at baseline and PK was noted.

### Pharmacodynamics

**Fig. 4:**
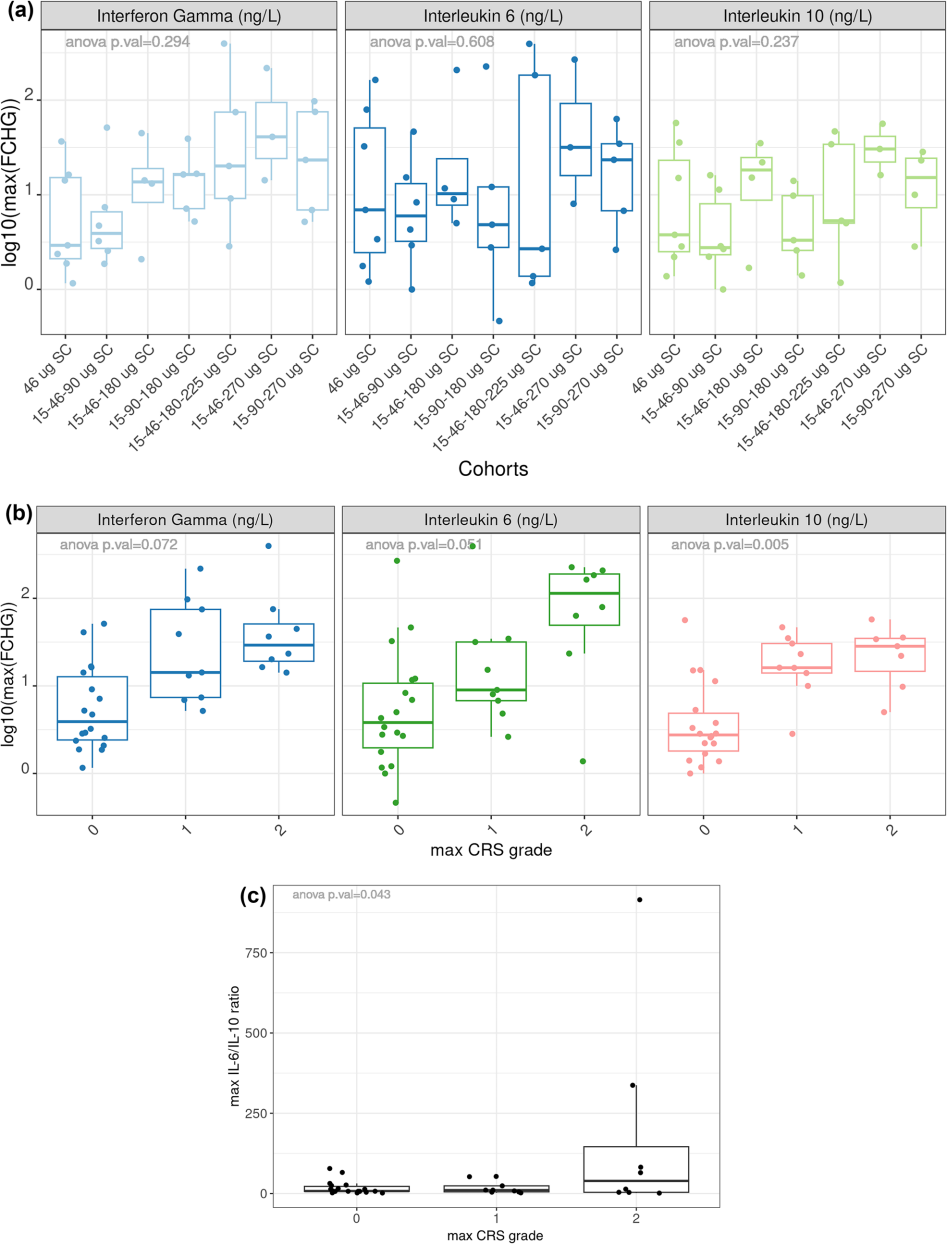
Cytokine induction following administration of JNJ-78306358: **a** Maximum fold change after treatment dose compared to baseline arranged by cohorts **b** Maximum fold change after treatment dose compared to baseline arranged by CRS grade **c** Comparison of IL-6 to IL-10 ratio arranged by CRS grade. **a** Box plot of maximum fold change (FCHG) of serum IFNγ, IL-6 and IL-10 across cohorts. Maximum fold change, shown in log10 scale, is defined as the highest fold change between post-treatment and baseline values across all sample collection timepoints. **b** Box plot of maximum fold change of serum IFNγ, IL-6 and IL-10 by CRS grade. No CRS is represented by grade 0. Maximum fold change, shown in log10 scale, is defined as the highest fold change between post-treatment and baseline values across all sample collection timepoints. **c** Box plot of maximum IL-6 to IL-10 ratio by CRS grade. Maximum ratio is defined as the highest ratio between IL-6 and IL-10 values across all sample collection timepoints. *CRS* cytokine release syndrome, *FCHG* fold change, *IL* interleukin

We observed an increase in JNJ-78306358-stimulated cytokine release during the study. Levels of interferon gamma (IFNγ) and to a lesser extent interleukin (IL)-6, and IL-10 increased with treatment dose (Fig. 4a), indicating JNJ-78306358 mediated T cell activation. We observed elevated cytokine levels (IL-6, IFNγ, and IL-10) in patients who had CRS (Fig. 4b). Notably, the pro-inflammatory (IL-6) to anti-inflammatory (IL-10) ratio was higher in grade 2 CRS compared to no CRS or grade 1 CRS (Fig. 4c).

**Fig. 5:**
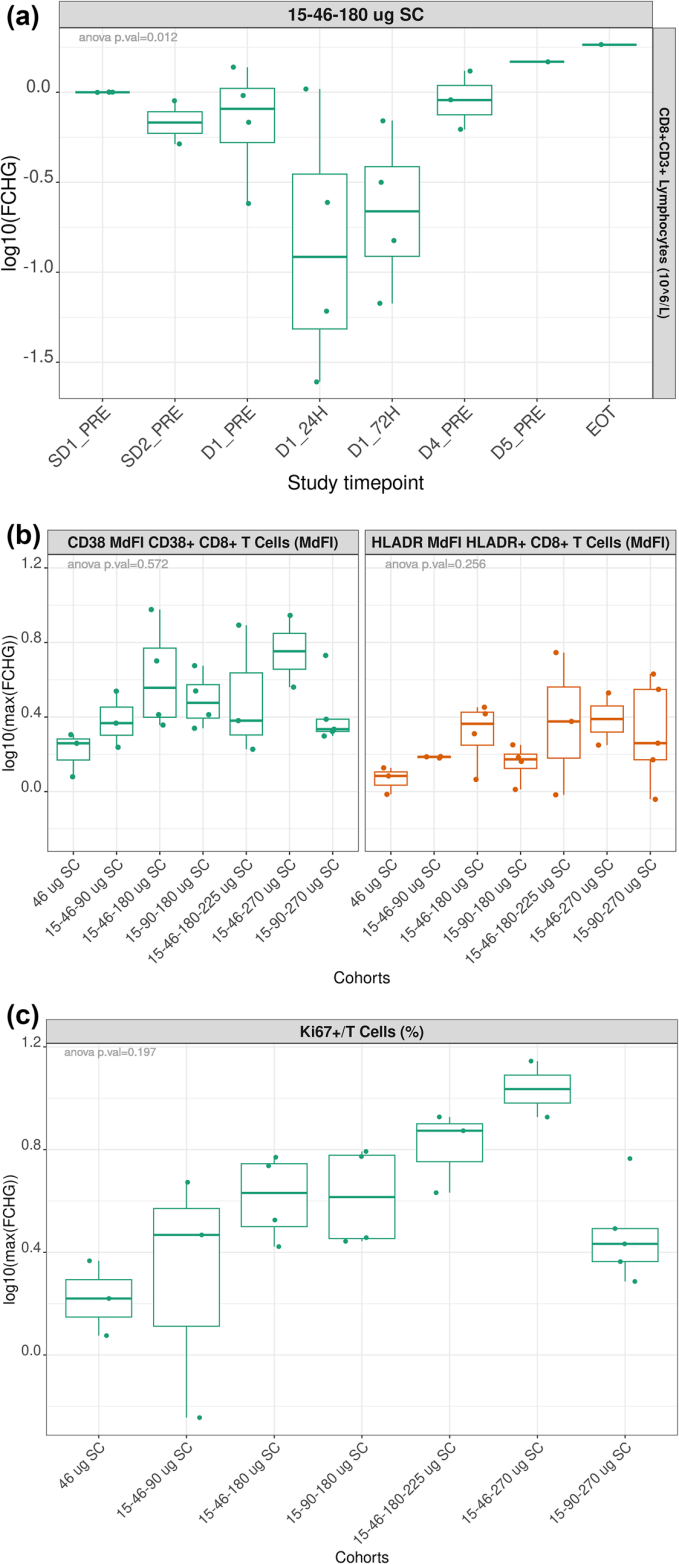
Effect of JNJ-78306358 on peripheral T cell migration, activation, and proliferation: **a** Induction of CD8 + T cell (CD8 + /CD3 +) margination by JNJ-78306358: Representative cohort of 15–48-180 ug **b** JNJ-78306358 induced activation of cytotoxic T cells (CD38 + /CD8 + and HLA-DR + /CD8 +) **c** T cell proliferation (Ki67 + /CD3 +) was induced by JNJ-78306358. **a** Longitudinal tracking of fold change (FCHG) of peripheral CD8 + /CD3 + T cell count compared to baseline levels in the 15–48-180 ug cohort. Fold change is presented in log10 scale. **b** Box plot of maximum fold change of CD38 + /CD8 + and HLA-DR + /CD8 + MdFI cohorts. Maximum fold change, shown in log10 scale, is defined as the highest fold change between post-treatment and baseline values across all sample collection timepoints. **c** Box plot of maximum fold change of the percentage of Ki67/CD3 + by treatment cohorts. Maximum fold change, shown in log10 scale, is defined as the highest fold change between post-treatment and baseline values across all sample collection timepoints. *CD* cluster of differentiation, *CRS* cytokine release syndrome, *EOT* end of treatment, *FCHG* fold change, *HLA-DR* human leukocyte antigen-DR isotype, *MdFI* median fluorescent intensity, *SC* subcutaneous

JNJ-78306358 treatment induced margination of CD8 + T cells from the periphery (Figs. 5a; S2); in contrast B cell numbers were not altered (data not shown). In addition, JNJ-78306358 stimulated peripheral T cell activation, as indicated by higher MdFI of the CD38 + and HLA-DR + in CD8 + T cells in post-treatment samples compared to baseline (Fig. 5b). CD4 + T cells were modestly activated compared to CD8 + T cells (Fig. S3). Additionally, higher T cell proliferation as indicated by increase in % Ki67 + /CD3 + cells was observed at higher treatment doses (Fig. 5c).

### Biomarkers

**Fig. 6:**
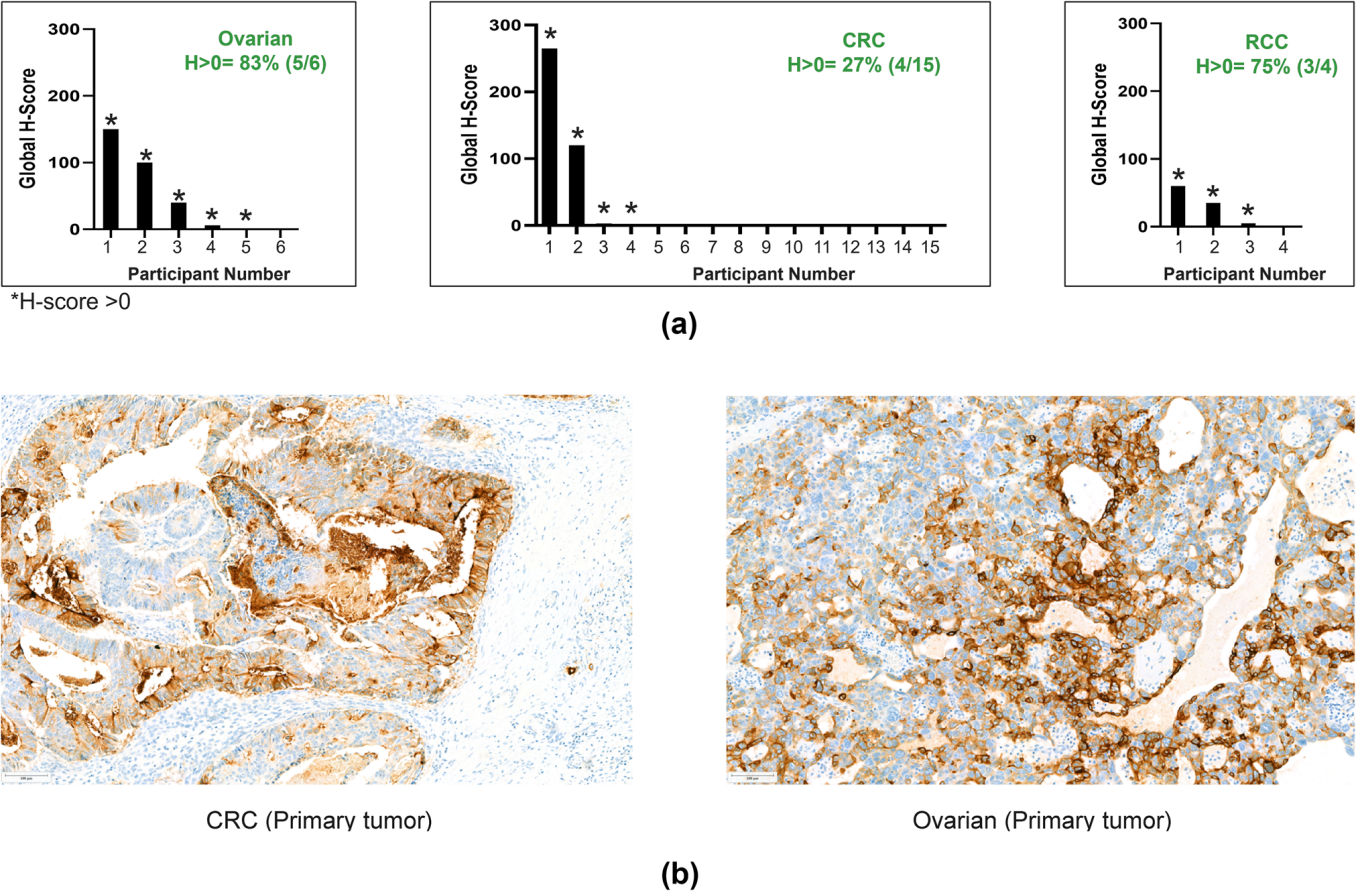
**a** Summary of HLA-G positivity (H-score > 0) by IHC in archival tissues **b** Representative HLA-G IHC images. *H-score > 0 **a** HLA-G levels in archival tumors was assessed by an IHC assay using 4H84 mAb. Global H-score from all IHC evaluable tumors is shown and separated by tumor types. Asterisk indicates samples with global H-score above 0. **b** Representative IHC images of HLA-G expression in archival tissues detected by the 4H84 mAb. *CRC* colorectal cancer, *HLA-G* human leukocyte antigen-G, *IHC* immunohistochemistry, *RCC* renal cell carcinoma

HLA-G was detected (IHC H-score > 0) in archival tissues from 12/25 evaluable patients (Fig. 6a): 3/4 (75.0%) patients with RCC, 4/15 (26.7%) patients with CRC, and 5/6 (83.3%) patients with OC (Fig. 6b). Of the 4 patients with target lesion reductions with evaluable archival tumor samples, only 2 had IHC H-scores > 0. No clear association between the levels of HLA-G expression in archival tissues and the extent of peripheral T cell activation was observed (Fig. S4).

## Discussion

Based on the results from both in vitro and preclinical studies [13], this first-in-human study aimed to assess the safety and preliminary antitumor activity of JNJ-78306358 in patients with advanced stage solid tumors with a high prevalence of HLA-G protein expression. The current study treatment showed limited efficacy, with 50% (17 of 34) of patients having SD, of which two patients had SD for > 40 weeks, though there were no objective responses (per RECIST Version 1.1 criteria). All patients discontinued the study treatment, largely due to progressive disease (82%).

The immune-associated toxicities may have prevented dose escalation of JNJ-78306358 to reach efficacious levels. In addition, the high frequency of ADA development reduced the drug exposure and had significant neutralization potential. Thus, the sponsor decided to terminate Part 1 dose escalation early and did not proceed to Part 2 dose expansion. As such, the limited enrolment data prevented drawing conclusions regarding the study objectives and endpoints, and no RP2D was established.

From the first dose level, immune-related toxicities were observed, particularly CRS events (none ≥ grade 3) and DLTs were observed in approximately 10% patients. CRS is a commonly recognized and frequent serious adverse event with bispecific T cell-engaging antibodies [18, 19]. High incidence of CRS has been noted with most of the currently FDA-approved bispecific T cell antibody therapies, such as blinatumomab, teclistamab-cqyv, mosunetuzumab and tebentafusp-tebn [18]. Step-up dosing and premedication with high dose corticosteroids were applied to mitigate CRS events. Step-up dosing partially mitigated CRS, as implementation of 15 ug and 46 ug step doses allowed for a treatment dose of 90 ug with only 1 grade 1 CRS event whereas 2 grade 2 CRS events were observed at the 46 ug dose level without step-up dosing. Of the patients who experienced ≥ 1 CRS events, 23% received tocilizumab treatment and this generally led to the rapid resolution of symptoms. This was in line with the recommendation that early intervention with tocilizumab is crucial during CRS events to prevent the development of severe and potentially life-threatening toxicities [20]. However, escalation to higher treatment doses was limited by CRS and CRS-associated toxicities. All the DLTs were preceded by a CRS event suggesting that they were related to CRS. Therefore, effective strategies to mitigate CRS are crucial for further promoting bispecific antibodies.

Although HLA-G expression is typically associated with the maternal–fetal interface, it is also upregulated in areas of inflammation [21–23], after viral infection [21, 24], and in response to certain cytokines [25]. The study excluded patients with chronic inflammatory conditions to avoid situations where HLA-G might be upregulated. However, it is unknown if preexisting inflammation or induced inflammation contributed to the observed CRS events or if an alternative mechanism contributed to CRS. In addition to the liver toxicities that met the DLT criteria, transient increases in liver enzymes were observed at multiple dose levels. Liver toxicities have been associated with CRS [26], but liver inflammation may possibly lead to upregulation of HLA-G in the liver. In a similar manner, preexisting or induced inflammation in the lungs may have contributed to observed pneumonitis. All patients enrolled in the study were heavily pretreated with chemotherapy and other agents which may have contributed to preexisting inflammation. Interestingly, patients with prior treatment with PD-(L1) inhibitors had a higher incidence and severity of CRS.

T cell-dependent bispecific therapies often induce a rapid and transient reduction in peripheral T cells (T cell margination) that in some cases associate with clinical response [27–29]. T cell margination is accompanied by T cell activation [30]. Consistent with prior findings, we observed peripheral T cell margination and T cell activation with increasing JNJ-78306358 treatment doses. Additionally, a dose dependent increase in serum cytokines (IFNγ, IL-6, and IL-10) was observed in this study. Collectively, immunophenotyping and cytokine data suggest JNJ-78306358 treatment elicits expected peripheral T cell activation responses. As on-treatment biopsies were not performed, it is unclear if T cell infiltration and activation occurs within the tumor. Limited T cell infiltration and suppressive tumor microenvironment are the main hurdles of CD3 redirecting therapies in solid tumors.

Excessive immune activation by T cell engagers can manifest in clinical CRS. In patients who experienced CRS, levels of these cytokines were found to be elevated, especially IL-6 and IFNγ. Induction of IL-6 in CRS is consistent with the use of tocilizumab as a mitigation strategy.

The selection of the tumor types included in this study was based on internal IHC staining of commercially acquired whole tissue samples with an α1-domain binding HLA-G antibody, 4H84 (12). As there is a low incidence of tumors expressing HLA-G isoforms lacking α3 domain, this preclinical prevalence data was determined to appropriately represent expression of HLA-G being targeted by JNJ-78306358. Given the caveats of the small sample numbers being assessed, prior treatment history, tumor heterogeneity and the IHC staining platform, the observed prevalence of HLA-G expression broadly agreed with preclinical data. HLA-G expression on archival tissues, which may not represent current tumor biology, did not correlate with target tumor reductions, prolonged stable disease, or the extent of peripheral T cell activity; however, such analyses are limited by the small sample size and inability to reach potentially efficacious doses.

ADA and soluble targets can act as sink that diminishes PK and drug efficacy. High incidence of ADA development (62.9%) noted during the study resulted in the loss of detectable PK; however, the impact of ADA could not be quantified due to low serum drug concentrations. The high incidence of ADA formation may be related to the SC route of administration that leads to high localized concentration of study drug and uptake by dendritic cells. Previous studies suggested that SC dosing is a preferable route of administration for bispecific antibodies for mitigating CRS and increasing the dose intensity [31, 32]. ADA formation that reduced serum drug concentrations has been observed for some other CD3-redirecting molecules given via the SC route [16, 33]; however, not all immune-targeting bispecific molecules that develop ADA have reduced serum concentrations [34]. Multiple forms of sHLA-G have been reported in serum from patients with cancer [35]. Preclinical modeling predicted serum levels of sHLA-G > 1 ng/ml to have impact on PK of JNJ-78306358 as it may act as a soluble sink [36]. The majority of the patient serum had no detectable sHLA-G, and the highest level observed was well below the expected threshold for target-mediated drug disposition effect. Additionally, no relationship was observed between baseline sHLA-G levels and PK. Collectively these data suggest that ADA, not sHLA-G, likely contributed to the diminished serum PK.

Taken together, the findings described herein are valuable to guide the selection of future bispecific antibody targets. Although targeting HLA-G via a CD3-redirecting mechanism with JNJ-78306358 did not prove effective therapy in this study due to inability to escalate to levels associated with efficacy in preclinical models, blocking HLA-G’s interaction with its receptors may still be a viable option [37]. Some success has been observed by targeting the immune inhibitory receptors, immunoglobulin-like transcript (ILT)-2 and ILT-4 [38–40]. Current findings contribute to the growing body of evidence that T cell-engaging therapies commonly lead to CRS events and patients benefit from tocilizumab as a supportive therapy [41].

## Supplementary Information

Below is the link to the electronic supplementary material.

Supplementary file1 (DOCX 483 kb)

## Data Availability

The data sharing policy of Janssen Pharmaceutical Companies of Johnson & Johnson is available at https://www.janssen.com/clinical-trials/transparency. As noted on this site, requests for access to the study data can be submitted through Yale Open Data Access [YODA] Project site at http://yoda.yale.edu.
