# Supplementary material for: Safety and clinical activity of JNJ-78306358, a human leukocyte antigen-G (HLA-G) x CD3 bispecific antibody, for the treatment of advanced stage solid tumors
Source: Cancer Immunol Immunother. 2024 Aug 6;73(10):205. doi: 10.1007/s00262-024-03790-7 (PMC11303617; doi:10.1007/s00262-024-03790-7)
Supplement: Supplementary file 1 — Supplementary file1 (DOCX 483 kb) [file 262_2024_3790_MOESM1_ESM.docx]

**SUPPLEMENT**

**Safety and Clinical Activity of JNJ-78306358, a Human Leukocyte Antigen-G
(HLA-G) x CD3 Bispecific Antibody, for the Treatment of Advanced Stage Solid Tumors**

Ravit Geva,^1*^ Maria Vieito,^2^ Jorge Ramon,^3^ Ruth Perets,^4^ Manuel Pedregal,^5^ Elena Corral,^3^ Bernard Doger,^5^ Emiliano Calvo,^3^ Jorge Bardina,^2^ Elena Garralda,^2^ Regina J. Brown,^6^ James G. Greger,^6^ Shujian Wu,^7^ Douglas Steinbach,^6^ Tsun-Wen Sheena Yao,^6^ Yu Cao,^8^ Josh Lauring,^6^ Ruchi Chaudhary,^6^ Jaymala Patel,^6^ Bharvin Patel,^6^ Victor Moreno^5^

**Fig. S1** Spider plot of percent change in the sum of diameters of target lesions overtime

| 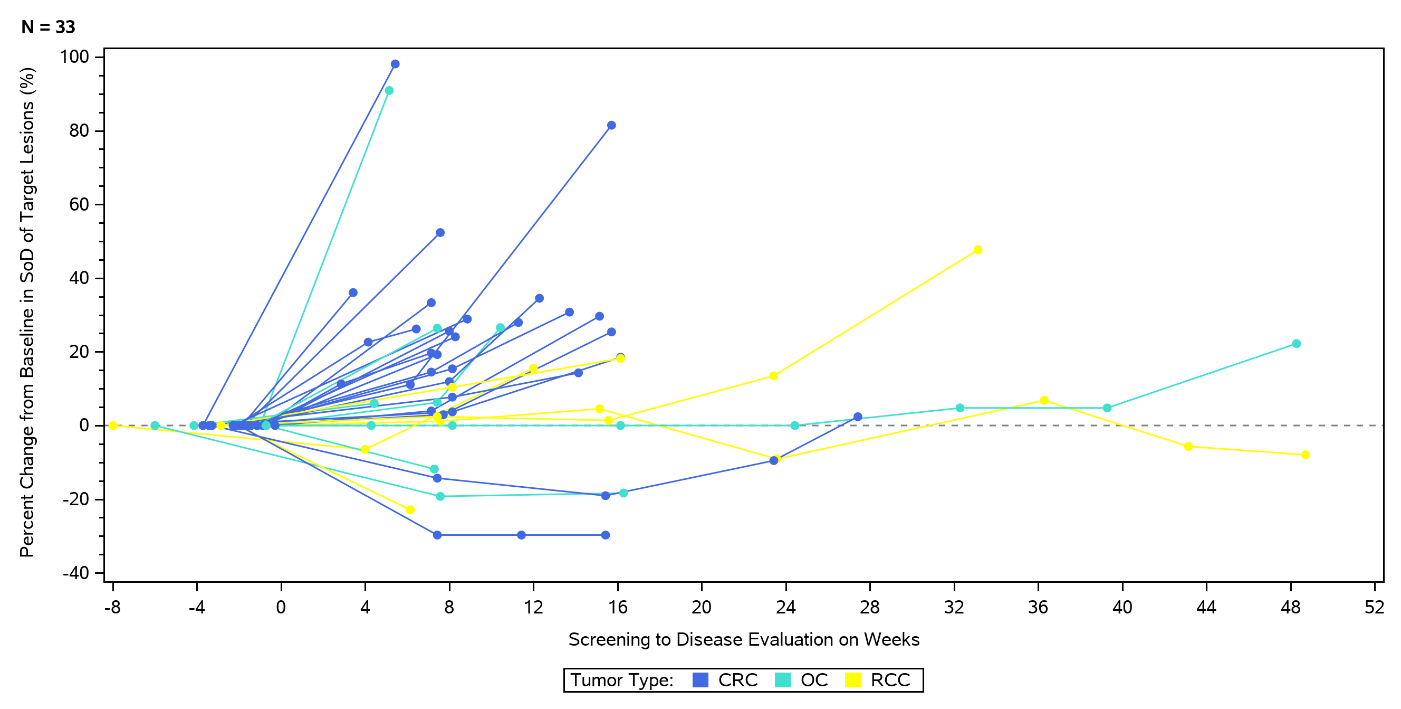 |
| --- |

Note: The number of subjects with baseline and at least one post-baseline assessment of all the tumors measured at baseline are shown.

*CRC* colorectal cancer, *OC* ovarian cancer, *RCC* renal cell carcinoma, *SoD* sum of diameters

**Fig. S2** Induction of CD4+ and CD8+ T cell margination by JNJ-78306358 by cohort


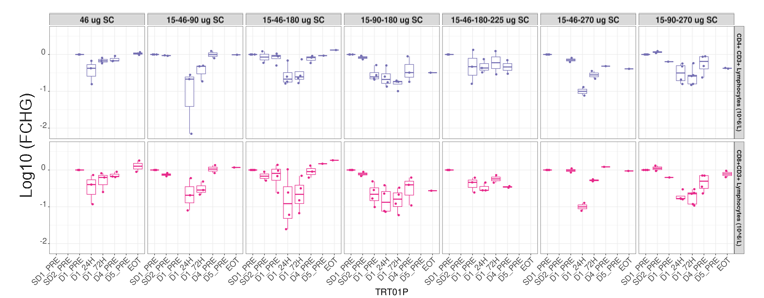


Note: Longitudinal tracking of fold change (FCHG) in peripheral CD4+/CD3+ (top) and CD8+/CD3+ (bottom) T cell count compared to baseline levels in all evaluable cohorts. Fold change is presented in log10 scale.

*CD* cluster of differentiation, *FCHG* fold change, *SC* subcutaneous

**Fig. S3** JNJ-78306358 induces modest activation of CD4+ T Cells

**
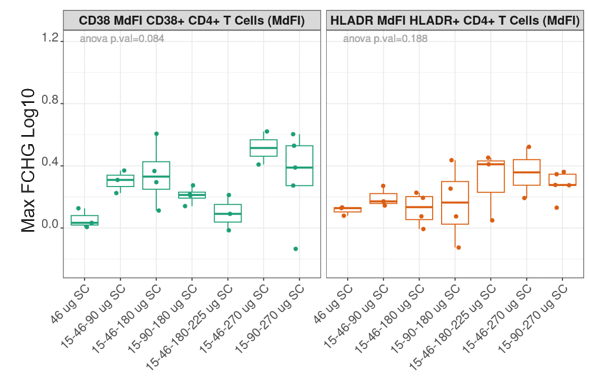
**

Note: Box plot of maximum fold change (FCHG) of CD38+ MdFI in CD38+ CD4+ and HLA-DR+ MdFI in HLA-DR+ CD4+ T cells compared to baseline by treatment cohorts. Maximum fold change, shown in log10 scale, is defined as the highest fold change between post-treatment and baseline values across all sample collection timepoints.

*CD* cluster of differentiation, *FCHG* fold change, *HLA-DR* Human Leukocyte Antigen-DR isotype, *MdFI*, median fluorescent intensity

**Fig. S4** Association between HLA-G expression in archival tissues and the levels of peripheral T cell activation or proliferation

**
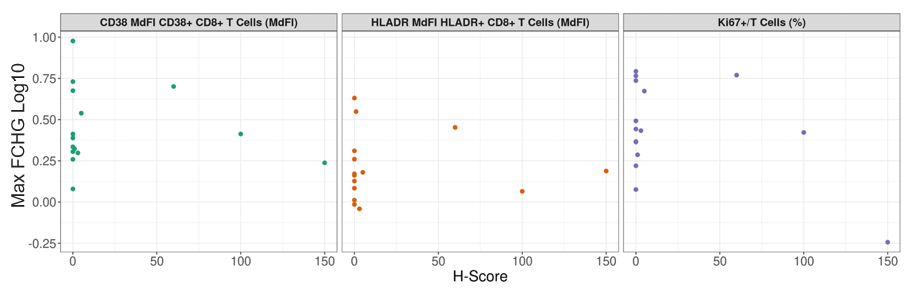
**

Note: Scatter plot of HLA-G H-score (from archival tumor tissues) and maximum fold change (FCHG) in T cell activation (CD38+/CD8+ MdFI, left; HLA-DR+/CD8+ MdFI, middle) and proliferation (percentage of Ki67/ CD3+ cells, right) levels. Maximum fold change, shown in log10 scale, is defined as the highest fold change between post-treatment and baseline values across all sample collection timepoints.

*CD* cluster of differentiation, *FCHG* fold change, *HLA-DR* Human Leukocyte Antigen-DR isotype, *MdFI* Median Florescence Intensity

**Table S1** Antibody clone information for flow cytometry analyses**​**

| Antibodies for TBNK Panel​ | | Antibodies for T Memory and Activation Panel (3-XD-1)​ | |
| --- | --- | --- | --- |
| Antibody | **Clone** | **Antibody** | **Clone** |
| CD3 FITC | SK7 | CD3 BUV395 | SK7 |
| CD16 PE | B73.1 | CD45 V500 | HI30 |
| CD56 PE | NCAM16.2 | CD4 BV711 | SK3 |
| CD45 PerCP-Cy5.5 | 2D1 (HLe-1) | CD8 BV650 | RPA-T8 |
| CD4 PE-Cy7 | SK3 | CD25 PE | 2A3 |
| CD19 APC | SJ25C1 | CD45RO BV605 | UCHL1 |
| CD8 APC-Cy7 | SK1 | CD127 BV785 | A019D5 |
|  |  | CCR7 (CD197) PE-Cy7 | G043H7 |
|  |  | FoxP3 APC | PCH101 |
|  |  | Ki67 FITC | B56 |
|  |  | HLA-DR PerCP-eFluor710 | L243 |
|  |  | ICOS (CD278) PE-eFluor610 | ISA-3 |
|  |  | CD38 BUV737 | HB7 |
|  |  | PD-1 (CD279) BV421 | EH12.2H7 |

**Table S2** Definition of Dose Limiting Toxicity (DLT)

| Hematologic Toxicity | |
| --- | --- |
| Neutrophil count decreased | Febrile neutropenia |
|  | Neutropenia: Grade 4 for ≥7 days |
| Platelet count decreased | Grade ≥3 thrombocytopenia with Grade ≥2 bleeding or any Grade 4 thrombocytopenia with duration ≥7 days |
| Any hematological toxicity | Fatal outcome |
| Non-hematological Toxicity | |
| Any non-hematological toxicity of Grade ≥3 or results in study drug discontinuation, with the following exceptions:   - Grade 3 fatigue, asthenia, fever, constipation, decreased appetite, nausea, vomiting, or diarrhea lasting ≤5 days with best supportive care - Grade ≥3 hypertension that can be controlled by medical management - Grade ≥3 ALT or AST that resolves to Grade ≤1 or baseline within 7 days, unless criteria for Hy’s law are met - Isolated Grade ≥3 alkaline phosphatase or GGT increase that returns to Grade ≤1 or baseline within 7 days - Grade ≥3 lipase or amylase increase not associated with clinical or radiological evidence of pancreatitis - Grade ≥3 electrolyte abnormalities that resolve spontaneously within 7 days, or that respond to best supportive care - Grade 3 sARR that resolves to Grade ≤1 within 48 hours - First occurrence of Grade 3 CRS | |

*ALP* alkaline phosphatase, *ALT* alanine aminotransferase, *AST* aspartate aminotransferase, *CRS* cytokine release syndrome, *DLT* dose-limiting toxicity, *GGT* gamma-glutamyl transferase; *sARR* systemic administration related reactions, *ULN* upper limit of normal
